# Effect of clenching on biomechanical response of human mandible and temporomandibular joint to traumatic force analyzed by finite element method

**DOI:** 10.4317/medoral.18488

**Published:** 2013-03-25

**Authors:** Kazuhiro Murakami, Kazuhiko Yamamoto, Tsutomu Sugiura, Masayoshi Kawakami, Yu B. Kang, Sadami Tsutsumi, Tadaaki Kirita

**Affiliations:** 1 DDS, PhD. Postdoctoral Fellow, Department of Oral and Maxillofacial Surgery, Nara Medical University, Kashihara City, Nara, Japan; 2 DDS, PhD. Associate Professor, Department of Oral and Maxillofacial Surgery, Nara Medical University, Kashihara City, Nara, Japan; 3 DDS, PhD. Assistant Professor, Department of Oral and Maxillofacial Surgery, Nara Medical University, Kashihara City, Nara, Japan; 4 PhD. Postdoctoral Fellow, Nihon University School of Dentistry, Tokyo, Japan 1-8-13, kandasurugadai, chiyoda, Tokyo, Japan; 5 PhD. Professor, Nihon University School of Dentistry, Tokyo, Japan; 6 DDS, DMSc. Professor and Chair, Department of Oral and Maxillofacial Surgery, Nara Medical University, Kashihara City, Nara, Japan

## Abstract

Purpose: The purpose of the present study was to analyze the effect of clenching on the biomechanical response of human mandible and temporomandibular joint (TMJ) to traumatic force by the finite element (FE) method. Material and Methods: FE models of the mandible and the TMJ in resting and clenching positions were prepared. Distribution and magnitude of von Mises stress were analyzed by applying force as a point load in the symphyseal, canine, body and angle regions of the mandible. In addition, strain energy density (SED) at the articular disc and in posterior connective tissue of TMJ was analyzed. Results: In the resting position, von Mises stress was mainly concentrated at the condylar neck and in the retromolar region of the mandible. In the clenching position, the stress at the condylar neck decreased in all loadings. The stress in the retromolar region similary decreased in the symphyseal, canine and body loading, respectively; however, higher stress was observed in the retromolar region on the loading side in the angle loading. High SED was generated at the articular disc and in posterior connective tissues of TMJ in the resting position. The SED in these tissues decreased in all loadings in the clenching position. Conclusions: Clenching generally reduces stress at the condylar neck and in the retromolar region of the mandible, and strain energy at the articular disc and in posterior connective tissue of TMJ by traumatic forces on the mandible; however, clenching induces greater stress in the retromolar region on the loading side by traumatic force to the angle region.

** Key words:**Mandibular, temporomandibular joint, traumatic force, clenching, finite element analysis.

## Introduction

Mandibular fracture is a common facial injury and is most frequently observed in the condylar region (1-3); however, in collision sports and assault, mandibular angle fracture also occurs at a high rate (4-10). The risk of mandibular angle fracture is primarily related to the presence of the third molar (8-10), but is also influenced by the occlusal condition. For most traumatic impacts, patients can respond to reduce the risk of severe injuries (11). One such response is clenching, since it can reduce stress at the site of impact and/or indirectly transmitted to vulnerable regions of the mandible by releasing it through the teeth; however, it is not easy to investigate the effect of clenching on the occurrence of mandibular fractures because of the complex structures and mechanical properties of the human mandible.

The finite element (FE) model is a non-invasive method to analyze the biomechanical response of bony specimens such as the human mandible (12-18). Several biomechanical studies have been performed focusing on the occurrence and osteosynthesis of mandibular fracture using the FE method (12-14). We also showed that the stress concentration area in the FE model is consistent with the fracture site of the human mandible in a tensile test (19); therefore, FE analysis is a useful tool to predict the biomechanical response of the mandible to various forces.

The purpose of the present study was to analyze the effect of clenching on the biomechanical response of the human mandible and the temporomandibular joint (TMJ) to traumatic force using the FE method by comparatively analyzing the stress concentration of the mandible and strain energy density (SED) development of the TMJ in resting and clenching positions.

## Material and Methods

-FE model

**Figure 1:**
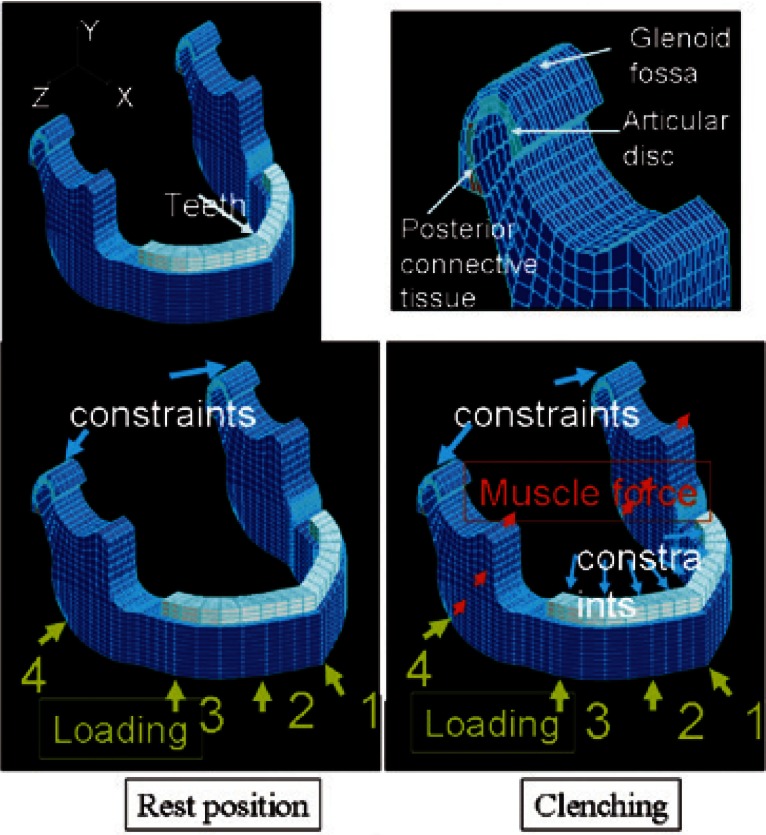
Finite element model of the mandible and the temporomandibular joint: Loading and boundary conditions in resting and clenching positions. Loading condition in resting position. For the boundary condition, the nodes of the upside of the bilateral glenoid fossa were constrained in all directions. A load with a magnitude of 980N was applied to the inferior border of the mandible, either in the symphyseal (loading 1), canaine (loading 2), body (loading 3) or angle (loading 4) region at a 45 degree angle from below the mandible. Loading condition in clenching position. For the boundary condition, the node of the upside of the bilateral glenoid fossa and upside of the whole dental arch were constrained in all directions. The proportion of muscle force magnitude was defined according to that in previous studies (16, 17). A Load with a magnitude of 980N was applied to the inferior border of the mandible, either in the symphyseal (loading 1), canaine (loading 2), body (loading 3) or angle (Loading 4) region at a 45 degree angle from below the mandible.

Three-dimensional FE models of the human mandible with the temporomandibular joint were constructed using COSMOS/M FE software (Structural Research and Analysis Corporation, Los Angeles, CA) and consisted of 8-node hexahedralsolid elements. The thickness of buccal and lingual cortical bone in the molar region was defined as 3.0 and 2.0 mm, respectively. The thickness of cancellous bone was defined as 10.0 mm and mandibular body height was defined as 25.0 mm according to the data determined by conebeam computed tomography of the human mandible in an adult man by Swasty et al (20). The teeth consisted of enamel and dentine. Periodontal membrane was not defined, since there was no significant difference with and without the periodontal membrane model in the previous study (21). The thickness of enamel was defined as 1.0 mm. The temporomandibular joint consisted of the glenoid fossa, the articular disc and the posterior connective tissue (Fig. 1). Models were composed of 12,510 elements and 14,934 nodes.

-Loading and boundary conditions

-Loading condition in resting position

For the boundary condition, the nodes of the upside of the bilateral glenoid fossa were constrained in all directions. A load with a magnitude of 980N was applied to the inferior border of the mandible, either in the symphyseal (Loading 1), canine (Loading 2), body (Loading 3) or angle (Loading 4) region at a 45 degree angle from below the mandible (Fig. 1).

-Loading condition in clenching position

For the boundary condition, the nodes of the upside of the bilateral glenoid fossa and upside of the whole dental arch were con-strained in all directions. The proportion of muscle force magnitude was defined according to previous studies (16,22). A load with a magnitude of 980N was applied to the inferior border of the mandible, either in the symphyseal (Loading 1), canine (Loading 2), body (Loading 3) or angle (Loading 4) region at a 45 degree angle from below the mandible (Fig. 1).

-Solution

The material properties were assumed to be homogeneous, isotropic, and linearly elastic. The material constants were defined as previously reported (23-27). The Poisson ratio and Young modulus were 0.3 and 1.4 ×104 MPa for cortical bone, 0.3 and 1.5 ×103 MPa for cancellous bone, 0.4 and 40 MPa for the articular disc, 0.48 and 8 MPa for posterior connective tissue, 0.3 and 5×104 MPa for enamel, and 1.07 ×104 MPa for dentin, respectively. Linear 3-dimensional FE analyses were performed. To evaluate mechanical stress in the mandible in resting and clenching positions, von Mises stresses were calculated. Furthermore, to evaluate the effect at the articular disc and in posterior connective tissue in the temporomandibular joint, axial strain, share strain, equivalent strain and strain energy density (SED) were calculated.

## Results

-Stress on the mandible in resting and clenching positions

**Figure 2:**
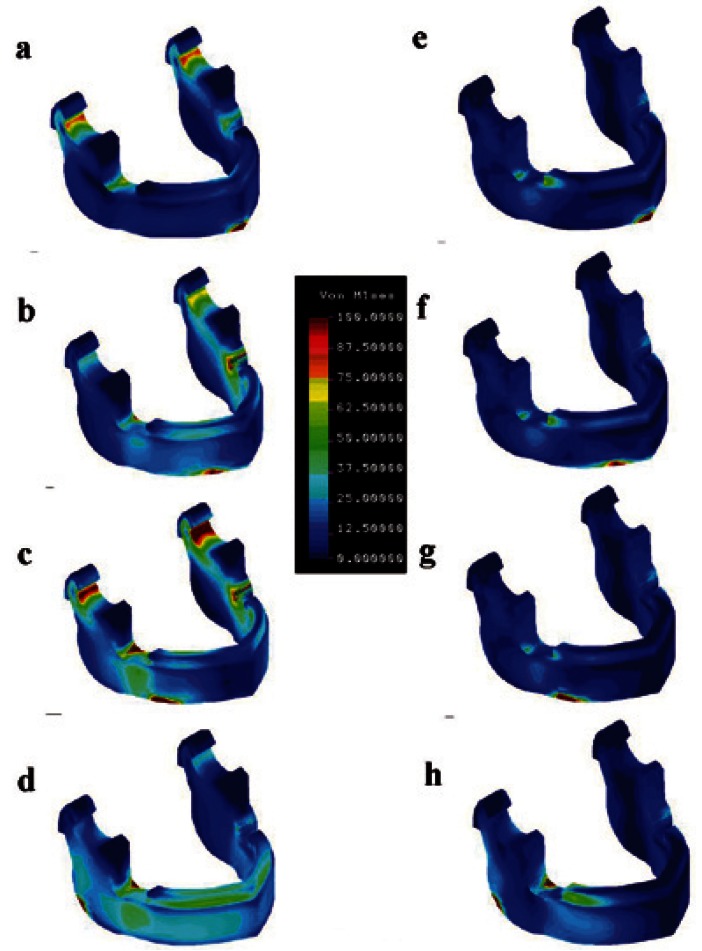
Distribution of von Mises stress in the mandible. a: Loading in symphyseal region in resting position (loading 1). b: Loading in canine region in resting position (loading 2). c: Loading in body region in resting position (loading 3). d: Loading in angle region in resting position (loading 4). e: Loading in symphyseal region in clenching position (loading 1). f: Loading in canine region in clenching position (loading 2). g: Loading in body region in clenching position (loading 3). h: Loading in angle region in clenching position (loading 4).

In FE model analysis in the resting position, the concentration of von Mises stress mainly occurred at the condylar neck and in retromolar regions. The maximal von Mises stresses in these regions are shown in  Table 1. In loading 1, stress of a similar magnitude was concentrated at the condylar neck and in the retromolar region and was almost equally distributed bilaterally; however, von Mises stress was greater in the retromolar region than at the condylar neck in loading 2-4. von Mises stress at the condylar neck was greater on the contralateral side of loading, and was greatest in loading 3 (141.5 MPa); however, the maximal von Mises stress in the retromolar region was greater on the loading side in loading 3 and 4, and was greatest in loading 3 (236.8 MPa). In the clenching position, von Mises stresses at the bilateral condylar neck decreased below 10 MPa. Stress in the retromolar region in loading 1- 3 also decreased below 51.2 MPa, but in loading 4, von Mises stress in the retromolar region on the loading side was 207.8MPa in the clenching position, which was greater than that in the resting position (141.7 MPa) (Fig. 2) ( Table 1).

**Table 1:**
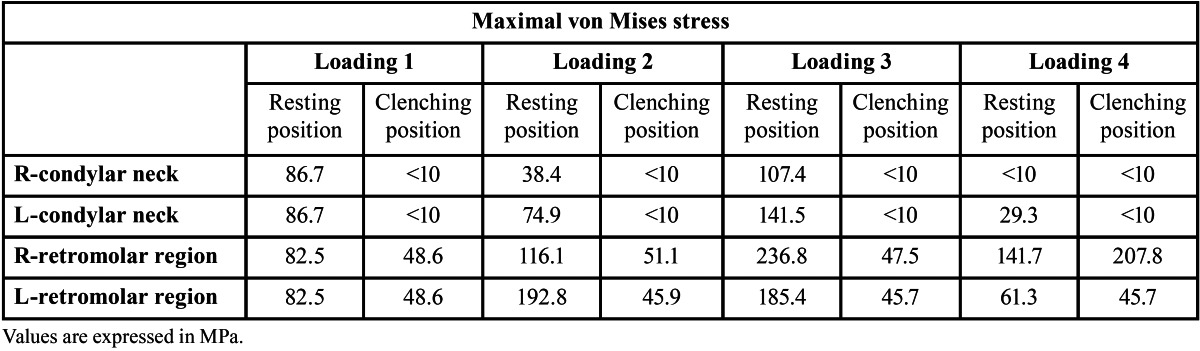
Maximal von Mises stress at the condylar neck and in the retromolar region in resting and clenching positions.

-Strain energy density (SED) in temporomandibular joint (TMJ)

**Figure 3:**
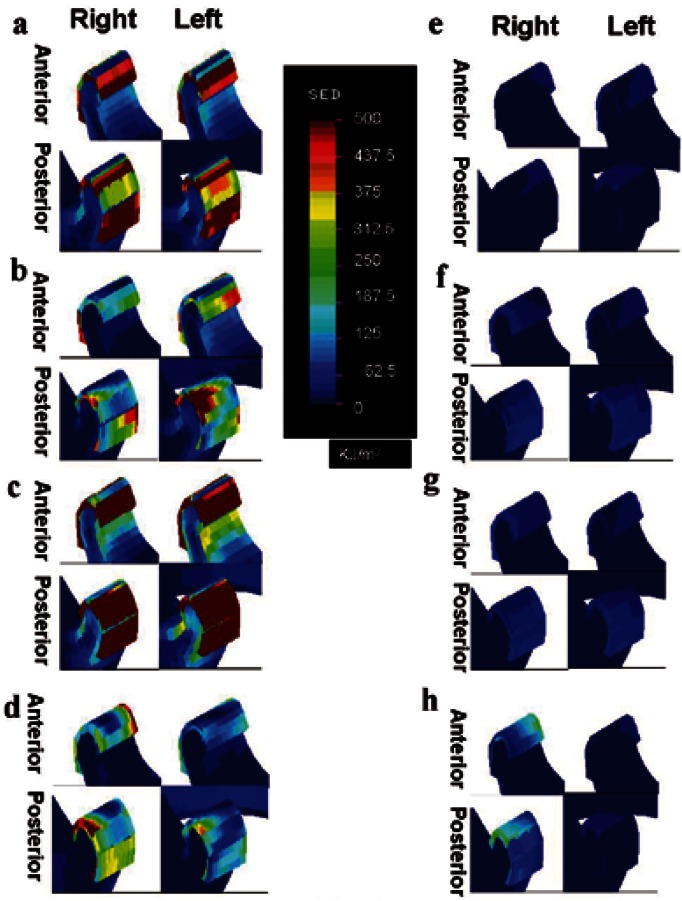
Distribution of strain energy density in the temoporomandibular joint. a: Loading in symphyseal region in resting position (loading 1). b: Loading in canine region in resting position (loading 2). c: Loading in body region in resting position (loading 3). d: Loading in angle region in resting position (loading 4). e: Loading in symphyseal region in clenching position (loading 1). f: Loading in canine region in clenching position (loading 2). g: Loading in body region in clenching position (loading 3). h: Loading in angle region in clenching position (loading 4).

High SED was generated at the articular disc and in posterior connective tissue in the resting position. The maximal SED in these regions is shown in  Table 2. A similar magnitude of SED, a little over 1100 KJ/m3, was bilaterally generated at the articular discs and in posterior connective tissue by loading 1. In loading 3, the magnitude of SED at the articular disc and in posterior connective tissue was more than 3000 KJ/m3. The maximal SED of 4719.3 KJ/m3 was observed in the right (loading side) posterior connective tissue in resting position in loading 3. In loading 2 and 3, the SED at the articular disc was a little greater on the contralateral side. In loading 4, the SED at the articular disc and in posterior connective tissue was greater on the loading side. In the clenching position, the SED in these tissues markedly decreased below 40 KJ/m3 in loading 1- 3. The SED at the articular disc and in posterior connective tissue on the loading side also decreased but by a lesser amount in loading 4 than in the other loadings (Fig. 3) ( Table 2).

**Table 2:**
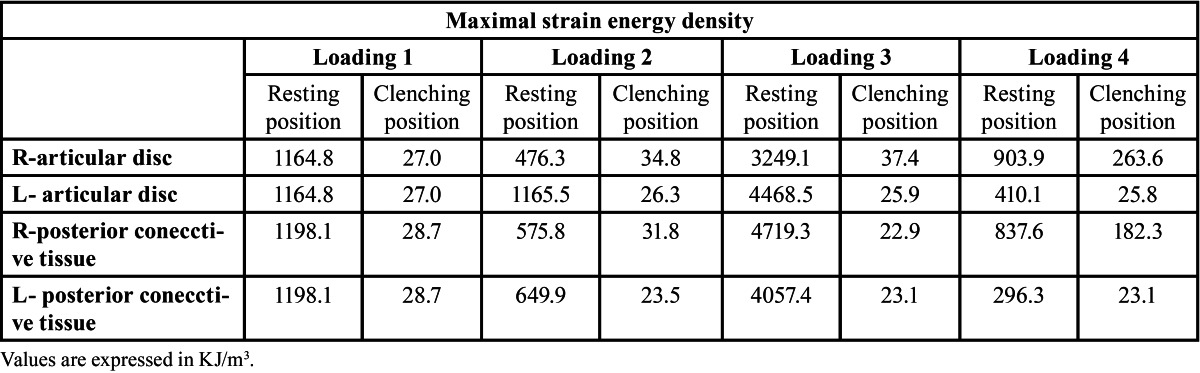
Maximal strain energy density at articular disc and in posterior connective tissue in resting and clenching positions.

## Discussion

Von Mises stress was mainly concentrated at the condylar neck and in the retromolar region of the mandible in a resting position by standardized traumatic force. Stress concentration at the condylar neck of the mandible is consistent with the fact that the condylar neck is the most common site of mandibular fracture (3,11). Since the condylar neck is the anatomically weakest region in the mandible, fractures may occur due to stress concentration by indirectly transmitted force. In the clenching position, however, the stress at the condylar neck markedly decreased. A similar protective effect of occlusion against condylar fracture was reported in a biomechanical study using the FE method (28). These findings suggest that clenching effectively reduces stress at the condylar neck and protects it from fracture.

Stress was also concentrated in the retromolar region in the resting position, which was greater than at the condylar neck, except in loading 1. High von Mises stress in the retromolar region, especially in loading 3, reflected the high share stress developed. Clinically, however, mandibular angle fracture primarily occurs by direct impact rather than by stress concentration by indirectly transmitted force (10). This discrepancy is probably ascribed to the volume of stress concentration relative to that of the entire angle region. Since the cross sectional area in the angle region is relatively large compared with that of the condylar neck, similar or even larger stress concentration localized in the retromolar region does not result in angle fracture. In the clenching position, stress in the retromolar region generally decreased; however, the greater stress developed in the retromolar region on the loading side in loading 4 (angle region). Such stress in addition to the impact directly applied to the angle region itself may render the mandible more susceptible to fracture.

The stress generated in soft tissue, such as the articular disc and posterior connective tissue, is very low compared with in the mandible and is difficult to examine; however, the strain generated in these soft tissues is very high. Therefore, we examined the SED at the articular disc and in posterior connective tissue by traumatic force. Maximal SED in the mandible was 1370 KJ/m3 in the right (loading side) retromolar region in loading 3 (loading in body region) in the resting position. Maximal SED at the articular disc and in posterior connective tissue was more than 3-fold that in the mandible under the same condition. This result indicates that the articular disc and posterior connective tissue in TMJ act as buffer material for the impact and have an important role in controlling strain distribution and reducing strain energy by impact to the mandible. In the clenching position, the SED in the TMJ region markedly decreased; therefore, clenching is considered to have a protective effect against traumatic injury in the TMJ region by releasing the stress through teeth.

There are inherent limitations of this study. The structures constructed as FE models were all assumed to be homogeneous and isotropic and to possess linear elasticity, although the cortical bone of the mandible is transversely isotropic and nonhomogeneous in living tissue. Furthermore, there is a difference in cortical thickness, bone density, and buccolingual width in the mandible. The force applied in this study was not dynamic, but static, although in previous studies, the stress distribution and magnitude by static analysis were almost consistent with those by dynamic analysis (28,29).

In conclusion, clenching generally reduces stress at the condylar neck and in the retromolar region of the mandible, and strain energy at the articular disc and in posterior connective tissue of the TMJ by traumatic forces to the mandible; however, clenching induces greater stress in the retromolar region on the loading side by traumatic force to the angle region.
